# Challenges in the diagnosis and management of depressive and cognitive disorders in older adults: a case report

**DOI:** 10.1590/1980-5764-DN-2025-0295

**Published:** 2025-06-02

**Authors:** Nour Regaieg, Dorra Mnif, Emna Smaoui, Fatma Guermazi, Salma Sakka, Ines Feki, Imen Baâti, Jawaher Masmoudi

**Affiliations:** 1 University of Sfax, School of Medicine of Sfax, Sfax, Tunisia.; 2 Hedi Chaker University Hospital, Psychiatry "A" Department, Sfax, Sfax Governorate, Tunisia.; 3 Habib Bourguiba University Hospital, Neurology Department, Sfax, Sfax Governorate, Tunisia.

**Keywords:** Dementia, Lewy Bodies, Depression, Cognition, Aged, Demência, Corpos de Lewy, Depressão, Cognição, Idoso

## Abstract

Dementia with Lewy Bodies and depression are two common conditions in older adults that can share clinical similarities or coexist. We aim to highlight the diagnostic and therapeutic challenges of these two pathologies through the case of Mrs. S., a 67-year-old patient admitted to the psychiatric department for agitation and insomnia. Her symptoms initially presented as depression, followed by severe agitation. In the admission interview, we observed an anxious patient with bradyphemia, primarily focused on well-detailed visual hallucinations related to death. Upon neurological examination, we identified Parkinsonian syndrome. The patient was treated with quetiapine 200 mg/day and sertraline 50 mg/day, leading to a marked improvement in both depressive and Parkinsonian symptoms. Ultimately, the diagnosis was a major depressive episode with psychotic and melancholic features as part of a bipolar II disorder. In neuropsychiatric presentations of dementia with Lewy Bodies, cognitive and neurological evaluations should always be considered. In some cases, clinical evolution remains the only means of distinguishing between depression and dementia with Lewy Bodies.

## RESUMO.

A demência por corpos de Lewy e a depressão são duas condições comuns em idosos e que podem partilhar semelhanças clínicas ou coexistir. Nosso objetivo é destacar os desafios diagnósticos e terapêuticos dessas duas patologias pelo caso da Sra. S., uma paciente de 67 anos de idade internada no Departamento de Psiquiatria por agitação e insônia. Inicialmente, ela apresentou sintomas de depressão seguidos de agitação severa. Na entrevista de admissão hospitalar, observamos uma paciente ansiosa com bradifemia, focada principalmente em alucinações visuais bem detalhadas relacionadas à morte. Após o exame neurológico, a Síndrome Parkinsoniana foi identificada. A paciente foi tratada com quetiapina 200 mg/dia e sertralina 50 mg/dia, levando a uma melhora acentuada dos sintomas depressivos e Parkinsonianos. Em última análise, o diagnóstico foi de transtorno depressivo maior com caraterísticas psicóticas e melancólicas como parte de um transtorno bipolar tipo II. Nas manifestações neuropsiquiátricas da demência por corpos de Lewy, as avaliações cognitivas e neurológicas sempre devem ser consideradas. Em alguns casos, a evolução clínica continua a ser o único meio de distinguir entre depressão e demência por corpos de Lewy.

## INTRODUCTION

Lewy Body Dementia (LBD) is the second most common neurodegenerative dementia after Alzheimer’s disease and is often underdiagnosed^1,2^. It is characterized pathologically by deposits of alpha-synuclein in the brain and encompasses three major clinical entities according to the classification proposed by Kosaka in 1980: Parkinson’s disease (PD), Parkinson’s disease with dementia (PDD), and dementia with Lewy Bodies (DLB)^3^. Early diagnosis of DLB can be challenging, particularly in psychiatric-onset presentations such as psychosis or mood disorders like depression^4,5^. Depression is a known risk factor for dementia and can also be a comorbidity or a frequent manifestation of DLB, with an estimated prevalence of 35%^6,7^. This prevalence is higher than in other dementias, including Alzheimer’s disease, making depression a supportive feature in the current clinical diagnostic criteria for DLB^1,6^. Therefore, identifying an underlying cognitive disorder in patients presenting with depression is essential for optimal therapeutic management. Depression negatively impacts the prognosis of DLB by causing distress, reducing quality of life, and exacerbating cognitive and functional impairment^1,8^. In this case report, we aim to illustrate the diagnostic and therapeutic challenges of managing both depression and cognitive impairment.

## CASE REPORT

We present the case of Mrs. S., a 67-year-old woman admitted to the psychiatric ward upon a third-party request due to severe anxiety, agitation, and insomnia. Mrs. S., the eldest of six siblings, had a primary school education, was married, and a mother of five children. She was a housewife living with her husband and children. She had no history of organic or psychiatric disorders and no family history of psychiatric illness. She did not use any particular drugs.

Her symptoms began 19 months before admission, following family stressors: her daughter’s divorce and the theft of her son’s work equipment. She became socially withdrawn, bedridden, refused to speak, lost her appetite, had severe insomnia, and neglected her hygiene. Over time, she exhibited excessive agitation, distractibility, and wandering behaviors. She spoke and laughed to herself, hid under sheets, behind doors, and in cupboards, or sometimes ran away from home, reporting visions of an evil woman attempting to kill her. She experienced sleep disturbances due to these visual hallucinations. In addition, she developed constipation and urinary incontinence.

Mrs. S. was seen by multiple psychiatrists and was prescribed various antipsychotics, including risperidone, olanzapine, aripiprazole, and amisulpride, with no noticeable improvement. Upon admission to our psychiatric unit, she was placed in a quiet room with strict monitoring of vital signs, a balanced diet, and oral rehydration. Laboratory tests showed mild anemia (hemoglobin: 10.6 g/dL) and thrombocytopenia (platelets: 114,000/mm^3^), while other parameters were normal.

During the admission interview, Mrs. S. displayed psychomotor slowing, reduced gestures, persistent anxiety, and bradyphemia. She covered herself with sheets, expressing delusions of persecution with well-formed, detailed visual hallucinations related to death. The neurological assessment was initially difficult. She was administered haloperidol (15 mg/day), orally, and clonazepam (3 mg/day). Two days later, upon reevaluation, an akinetic-rigid Parkinsonian syndrome was identified, with an initial Unified Parkinson’s Disease Rating Scale (UPDRS) score of 25, indicating a severe extrapyramidal syndrome. Temporal disorientation was verified upon cognitive assessment, but objective memory loss was not found in the Mini-Mental State Examination (MMSE). Other cognitive functions, notably visuospatial apraxia, were difficult to assess due to the patient’s distractibility and the Parkinsonian syndrome.

Two main differential diagnoses were considered: DLB and major depressive episode with psychotic features as part of a bipolar II disorder. DLB was suspected given the advanced age, neuropsychiatric symptoms including depressive symptoms, detailed visual hallucinations, lack of response to antipsychotics, constipation, urinary incontinence, and rapid worsening of Parkinsonian symptoms with classic antipsychotics. Conversely, the diagnostic of major depressive disorder with psychotic features was proposed due to the presence of symptoms suggesting depression, such as social isolation, mutism, clinophilia, self-neglect, anorexia, and insomnia, which followed stress factors, along with psychotic symptoms characterized by a delusion of persecution and visual hallucinations. The bipolar II disorder was considered due to history of episodes of heightened mood indicative of hypomanic episodes.

Thyroid, immune, and paraneoplastic tests were negative. Brain MRI and EEG were normal. Functional imaging (SPECT scan) and polysomnography, which could further guide diagnosis, were unavailable in our hospital. Haloperidol was discontinued, and quetiapine was initiated at 25 mg/day, which gradually increased to 200 mg/day. Over time, the patient showed significant improvement in anxiety, hallucinations, sleep, and appetite, with resolution of Parkinsonian symptoms.

Upon subsequent psychiatric evaluations, we found a prominent depressive syndrome characterized by psychomotor slowing, reduced facial expressiveness, depressed mood, and bradyphrenia. Mrs. S. spoke softly, expressing sadness related to traumatic life experiences, including the sudden death of her father and her responsibility for her siblings after her mother’s early death. Some visual hallucinations with death-related themes persisted. The diagnostic of a major depressive episode with psychotic and melancholic features as part of a bipolar II disorder was confirmed. A decision was made to introduce sertraline, starting at 25 mg/day and increasing it to 50 mg/day. One month later, depressive symptoms improved significantly, Parkinsonian symptoms resolved completely, and cognitive reassessment was normal.

## DISCUSSION

In this case, clinical evolution played a crucial role in diagnostic orientation. Ultimately, DLB was deemed unlikely, and the final diagnosis was a major depressive episode with psychotic and melancholic features.

DLB is characterized by fluctuating cognitive impairment, recurrent, well-formed visual hallucinations, and Parkinsonism, which manifests as rigidity, resting tremor, bradykinesia, gait disturbances, or postural instability. Cognitive deficits, including memory impairment, attention deficits, and visuospatial dysfunction, typically emerge later in the disease course. REM (rapid eye movement) sleep behavior disorder, confirmed by polysomnography, is another important feature^4,9^.

Neuropsychiatric symptoms, particularly apathy, depression, and psychosis, are common in the prodromal stage of DLB, resulting in behavioral disturbances such as visual hallucinations, delusions, agitation, and anxiety^3,10^. Depression is particularly prevalent, affecting 30–60% of DLB patients^11–14^. These neuropsychiatric manifestations are linked to neurohormonal imbalances involving dopamine, serotonin, noradrenaline, and acetylcholine^15,16^. Additional mechanisms include α-synuclein pathology, synaptic zinc dysregulation, proteasome inhibition, and gray matter loss in prefrontal and temporal areas^7,17^. Depression in DLB may also result from atrophy or dysfunction of the pontomesencephalic-limbic emotional circuitry^17^.

Parkinsonian syndrome in DLB is associated with dopaminergic neuronal loss, leading to impaired voluntary movement facilitation^18,19^. In this case, normal EEG and brain MRI did not exclude DLB, as structural changes may only appear a decade after disease onset. Functional imaging could have provided further diagnostic clarity, particularly showing occipital hypometabolism associated with DLB visual hallucinations^9^.

The initial diagnostic uncertainty made treatment selection challenging. Antipsychotic treatment in suspected DLB can paradoxically worsen motor symptoms due to heightened antipsychotic sensitivity, particularly with first-generation agents such as haloperidol^3,20^. Atypical antipsychotics, particularly quetiapine and clozapine, are preferred due to their lower affinity for dopaminergic receptors^20,21^. In our case, quetiapine was chosen based on the patient’s anemia and thrombocytopenia, which contraindicated clozapine as well as for its effect both as a thymoregulator and an antipsychotic in bipolar depression.

The recommended quetiapine dose for DLB is 25–400 mg/day and has been associated with long-term symptom improvement^5^. In bipolar depression of older adults, quetiapine and lurasidone monotherapy are recommended as first-line options for the acute management. Conversely, antidepressants, such as selective serotonin reuptake inhibitors (SSRIs), could be used in combination with mood stabilizers in patients who do not tolerate or do not respond to other treatments^22^. SSRIs are consistently preferred over tricyclic antidepressants due to their better tolerability^23^. Given the severity of depressive symptoms in our case, we have chosen to associate sertraline in order to potentiate the effect of quetiapine.

In conclusion, major depressive episodes in older adults can present atypically, complicating differential diagnosis. Diagnostic uncertainty should not delay treatment initiation. Medication selection should consider associated somatic symptoms to ensure better tolerance. In the absence of highly sensitive and specific biomarkers, clinical evolution remains crucial for diagnosis. This case highlights the importance of cognitive evaluation and careful follow-up in older patients with psychiatric symptoms to detect possible underlying dementia and adjust treatment accordingly.
